# Stroke pathway performance assessment: a retrospective observational study

**DOI:** 10.1186/s12913-023-10343-8

**Published:** 2023-12-11

**Authors:** Jacopo Camporesi, Silvia Strumia, Andrea Di Pilla, Matteo Paolucci, Diego Orsini, Chiara Assorgi, Maria Gabriella Cacciuttolo, Maria Lucia Specchia

**Affiliations:** 1 Intensive Care Unit (ICU) Morgagni-Pierantoni Hospital, AUSL Romagna, Forlì, Italy; 2 Neurology Unit, Morgagni-Pierantoni Hospital, AUSL Romagna, Forlì, Italy; 3 Direzione Sanitaria, Azienda Ospedaliera San Camillo-Forlanini, Roma, Italy; 4 Alta Scuola di Economia e Management dei Sistemi Sanitari, Università Cattolica del Sacro Cuore, Roma, Italy; 5 Neurology Unit Forlì-Cesena, AUSL Romagna, Forlì, Italy; 6 IRCCS Istituto delle Scienze Neurologiche di Bologna, Bologna, Italy; 7 Dipartimento di Scienze della Vita e Sanità Pubblica—Sezione di Igiene, Università Cattolica del Sacro Cuore, Roma, Italy; 8 Fondazione Policlinico Universitario “A. Gemelli” IRCCS, Roma, Italy

**Keywords:** Stroke pathway, Pharmacological thrombolysis, COVID-19, Performance assessment

## Abstract

**Background and Aim:**

Performance assessment of the Stroke Pathway is a key element in healthcare quality. The aim of this study has been to carry out a retrospective assessment of the Stroke Pathway in a first level Stroke Unit in Italy, analyzing the temporal trend of the Stroke Pathway performance and the impact of the COVID-19 pandemic.

**Methods:**

A retrospective observational study was carried out analyzing data from 1/01/2010 to 31/12/2020. The following parameters were considered: volume and characteristics of patients with ischemic stroke undergoing intravenous thrombolysis, baseline modified Rankin Scale (mRS) and National Institutes of Health Stroke Scale (NIHSS) scores, Onset-to-Door (OTD), Door-To-Imaging (DTI) and Door-To-Needle (DTN) Times, mRS score 3 months after the ischemic event onset (3 m-mRS) and NIHSS score 24 h after the ischemic event onset (24 h-NIHSS). The study also compared the pre-COVID-19 pandemic period (March-December 2019) with the one immediately following it (March-December 2020).

**Results:**

418 patients were included. Over time, treatment was extended to older patients (mean age from 66.3 to 75.51 years; *p = 0.006*) and with a higher level of baseline disability (baseline mRS score from 0.22 to 1.22; *p = 0.000*). A statistically significant reduction over the years was found for DTN, going from 90 min to 61 min (*p = 0.000*) with also an increase in the number of thrombolysis performed within the “golden hour” – more than 50% in 2019 and more of 60% in 2020. Comparing pre- and during COVID-19 pandemic periods, the number of patients remained almost unchanged, but with a significantly higher baseline disability (mRS = 1.18 vs. 0.72, *p = 0.048*). The pre-hospital process indicator OTD increased from 88.13 to 118.48 min, although without a statistically significant difference (p = 0.197). Despite the difficulties for hospitals due to pandemic, the hospital process indicators DTI and DTN remained substantially unchanged, as well as the clinical outcome indicators 3 m-mRS, NHISS and 24 h-NHISS.

**Conclusions:**

The results of the retrospective assessment of the Stroke Pathway highlighted its positive impact both on hospital processes and patients’ outcomes, even during the COVID-19 pandemic, so that the current performance is aligning itself with international goals. Moreover, the analysis showed the need of improvement actions for both hospital and pre-hospital phases. The Stroke Pathway should be improved with the thrombolysis starting in the diagnostic imaging department in order to further reduce the DTN score. Moreover, health education initiatives involving all the stakeholders should be promoted, also by using social media, to increase population awareness on timely recognition of stroke signs and symptoms and emergence medical services usage.

## Introduction

Stroke is a clinical syndrome characterized by the sudden onset of a focal neurological deficit (sometimes complete) persisting for more than 24 h or leading to death. The 24-hour limit is arbitrary and, according to some definitions, it should be replaced by neuroimaging data (i.e. if a brain injury area is visualized, it should be considered a stroke even if symptoms have lasted less than 24 h) [1].

Every year worldwilde, according to the World Health Organization (WHO), 15 million people are affected by stroke. Of these, 5 million die (in Europe, every year, there are about 650,000 deaths due to stroke) and another 5 million have a permanent disability [2]. Stroke incidence increases with age. From 55 years, it doubles every decade and 95% of all strokes occur in people over 65 years. According to the Global Burden of Disease, in Italy, the age-standardized incidence of stroke is 64-85.5 cases/100,000 inhabitants, with a decrease of 22% compared to 1990 [3, 4].

The main modifiable risk factors are tobacco smoking, alcohol consumption, poor diet, physical inactivity, and high blood pressure [5–7].

Stroke can be ischemic or hemorrhagic [8]. Ischemic stroke is the main stroke subtype (62.4% of total cases) and, in recent years, a decrease of its incidence and mortality has been registered, thanks to the improvement of preventive strategies (risk factors control) and therapeutic approaches [3, 8]. Concerning the latter, the two main treatments available are intravenous thrombolysis and mechanical thrombectomy, whose effectiveness, in terms of brain damage, mortality and morbidity reduction, is time-dependent [8].

In 2015, the Italian regulation, with the Decree n.70 of the Ministry of Health, identified a Stroke Pathway, that is a patient-centered and evidence-based logical sequence (from a spatial and organizational view point) of activities aimed at guaranteeing the best approach to the clinical management of stroke, optimizing patient outcomes and maximizing clinical efficiency [9, 10]. The Stroke Pathway is based on 3 stages: pre-hospital, hospital and post-hospital phase. The pre-hospital phase is the time interval between the onset of symptomatology and the arrival to the Emergency Department (ED). The hospital phase consists of patient assessment, diagnosis and treatments and the post-hospital phase of the individual rehabilitation project. The Decree n.70 also defines the standards of the Hospital units for the treatment of patients with acute cerebral stroke (first and second level Stroke Units) [9].

The performance assessment of the Stroke Units is a key element in the process of continuous healthcare quality improvement. The SITS-MOST (Safe Implementation of Thrombolysis in Stroke - Monitoring Study) registry is a world-leading platform for stroke data of more than 200,000 patients from more than 1600 Stroke Units. The network currently consists of over 3000 stroke professionals from over 70 countries. The registry serves as a tool for structured data entry and allows for national and international benchmarking of treatment outcomes. All patients undergoing pharmacological thrombolysis in the Stroke Units must be recorded on the registry [11].

The outbreak of the Coronavirus disease 2019 (COVID-19) pandemic heavily impacted the Italian healthcare system. The pre-hospital and hospital performance of integrated care pathways focused on time-dependent diseases such as stroke has been affected by the pandemic [12].

The aim of this study has been to carry out a retrospective assessment of the Stroke Pathway in a first level Stroke Unit in Northern Italy, particularly focusing on intravenous thrombolytic therapy. The specific objectives have been to analyze the temporal trend of the Stroke Pathway performance and the possible impact of the COVID-19 pandemic.

## Methods

This study was approved by the Ethical Committee of the Romagna Scientific Institute (Protocol number 0006510); It was carried out in accordance with the Helsinki Declaration and EU Regulation 2016/679 (GDPR).

To analyze the temporal trend of the Stroke Pathway performance, a retrospective observational study was carried out analyzing data from 1/01/2010 to 31/12/2020 collected in the SITS-MOST (Safe Implementation of Thrombolysis in Stroke - Monitoring Study) registry, the global web registry of all patients undergoing pharmacological thrombolysis [11]. Patients diagnosed with ischemic stroke, who were considered eligible for treatment with pharmacological thrombolysis at the Morgagni Pierantoni Hospital in Forlì (Emilia Romagna Region, Italy) over the ten-year period covered by the analysis, were included in the study.

The trends of the following variables were analyzed:

- Number (case volume) and demographic characteristics of patients with ischemic stroke undergoing intravenous thrombolysis;
- Onset-To-Door time – OTD: time interval between the onset of symptomatology and the arrival to ED (*process indicator* related to the *pre-hospital* phase of the Stroke Pathway) [13];
- Door-To-Imaging time – DTI: time interval between the arrival to ED and the starting of Imaging (computed tomography (CT)/magnetic resonance (MR)) (*process indicator* related to the *hospital phase* of the Stroke Pathway) [13];
- Door-To-Needle time – DTN: time interval between the arrival to ED and the starting of pharmacological thrombolysis (*process indicator* related to the *hospital phase* of the Stroke Pathway) [13];
- modified Rankin Scale (mRS) and National Institutes of Health Stroke Scale (NIHSS) scores at the baseline (first) patient’s assessment (*clinical outcome indicators*). The mRS is the most widely used clinical outcome parameter for measuring the degree of disability or dependence in the daily activities of people who have suffered a stroke or other causes of neurological disability [14]. The NIHSS is a tool used to objectively quantify the stroke severity and the related impairment [15].
- mRS [14] score 3 months after the ischemic event onset (3 m-mRS) and NIHSS [15] score 24 h after the ischemic event onset (24 h-NIHSS).

For each variable, mean and standard deviation were calculated.

The statistical analysis was carried out by performing an Analysis of Variance (ANOVA).

To analyze the possible COVID-19 impact on the Stroke Pathway performance, the study also compared, for the same parameters, the pre-COVID-19 pandemic period (March-December 2019) with the one immediately following it (March-December 2020), through a Student t-Test. The statistical significance was set at p ≤ 0.05. The *SPSS* software *v.25* was used.

## Results

**Fig. 1:**
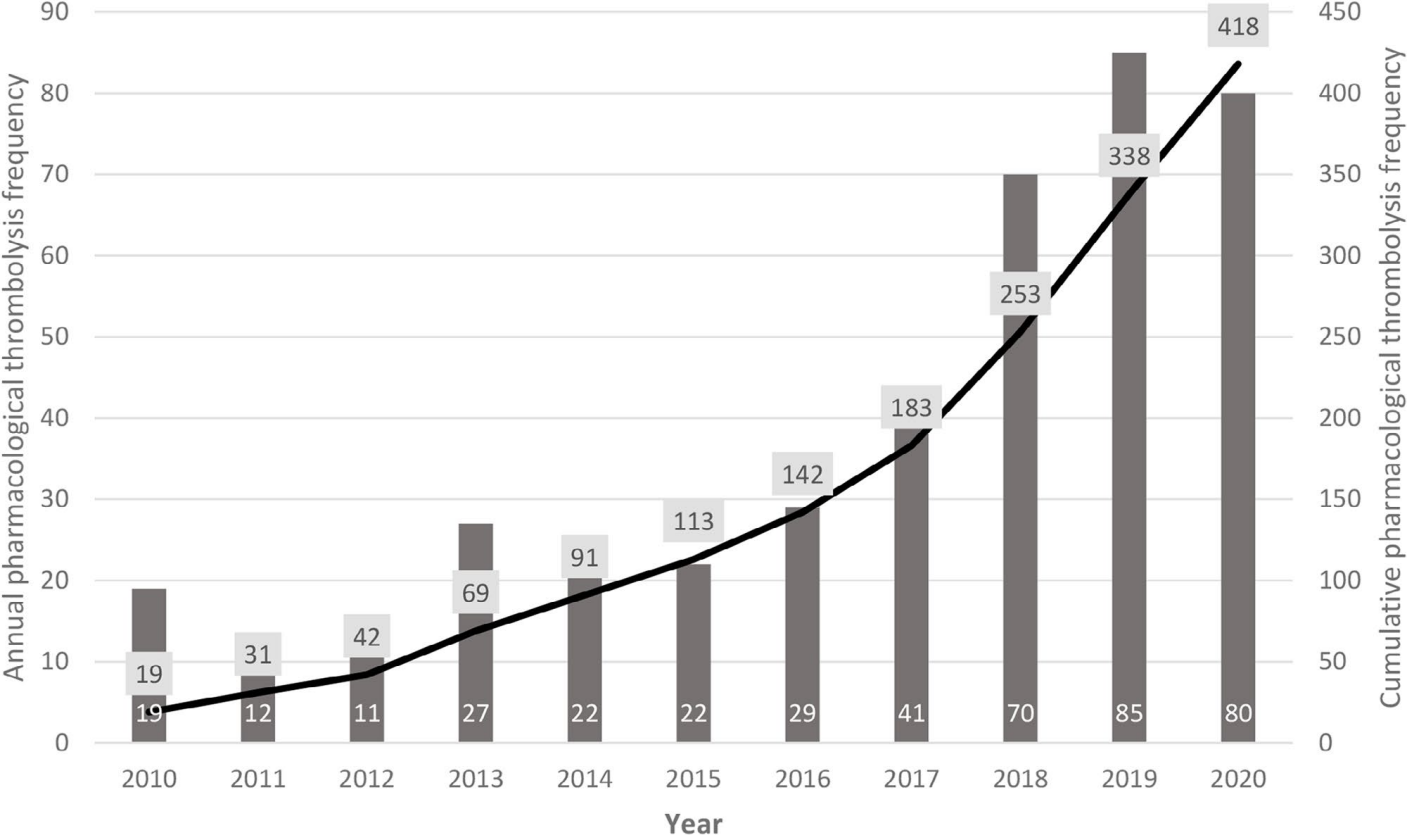
Pharmacological thrombolysis annual and cumulative frequency

The main study results over the 10-year period considered are reported in Table 1. A total number of 418 patients diagnosed with ischemic stroke was considered eligible for treatment with pharmacological thrombolysis. As for the treatment annual and cumulative frequency, 56% of patients were treated during the last three years considered (1 January 2018–31 December 2020) (Fig. 1).

**Table 1:** Stroke pathway performance temporal trend

		2010	2011	2012	2013	2014	2015	2016	2017	2018	2019	2020	Total	*p*
Number of patients		19	12	11	27	22	22	29	41	70	85	80	418	
%		4.5	2.9	2.6	6.5	5.3	5.3	6.9	9.8	16.7	20.3	19.1	100.0	
Cumulative %		4.5	7.4	10.0	16.5	21.8	27.0	34.0	43.8	60.5	80.9	100.0		
Gender	M	13	5	6	11	13	8	17	18	29	45	35	200	
	F	6	7	5	16	9	14	12	23	41	40	45	218	
Age at stroke onset	Mean	66.32	66.75	65.73	71.07	75.91	70.05	70.90	68.44	74.36	75.72	75.51	72.86	***0.006***
	Standard deviation	10.562	15.639	11.628	9.575	8.088	14.844	11.261	15.694	13.403	14.548	14.303	13.744	
Onset-to-Door (OTD)	Mean	67.53	76.83	66.60	72.87	68.00	65.95	80.32	82.31	76.14	84.70	111.74	83.60	*0.275*
	Standard deviation	29.879	34.274	40.344	47.276	37.289	41.755	44.811	50.909	42.209	73.098	155.576	83.049	
Door-to-Imaging CT/MR (DTI)	Mean	19.50	23.27	32.11	31.24	33.90	34.05	37.05	39.38	38.89	33.86	31.64	33.78	*0.777*
	Standard deviation	11.838	11.001	18.496	9.736	21.682	19.476	15.145	21.226	69.009	24.009	29.177	35.586	
Door-to-Needle (DTN)	Mean	90.26	79.42	99.10	84.33	102.50	73.23	75.16	65.08	64.09	63.12	61.44	70.54	***0.000***
	Standard deviation	44.369	25.123	26.510	27.926	34.942	30.046	30.454	24.534	28.270	29.430	25.647	31.254	
Modified Ranking Scale (mRS) (Baseline)	Mean	0.22	0.17	0.00	0.30	0.64	0.50	0.41	0.55	1.09	0.74	1.22	0.74	***0.000***
	Standard deviation	0.732	0.577	0.000	0.724	1.136	0.913	0.907	1.037	1.358	1.156	1.499	1.207	
NHISS	Mean	11.11	11.58	9.73	12.22	10.45	12.09	10.26	7.50	10.43	10.23	10.42	10.34	*0.283*
	Standard deviation	5.635	4.420	4.819	6.571	5.134	5.756	4.840	6.552	6.959	7.315	7.402	6.582	
Modified Ranking Scale after 3 months (mRS 3 m)	Mean	3.13	2.58	2.45	2.63	3.18	2.41	1.71	2.33	2.95	2.63	2.46	2.59	*0.517*
	Standard deviation	2.363	2.392	2.423	2.483	2.462	2.239	2.106	2.030	2.064	2.304	2.465	2.272	

Concerning patients’ characteristics, during the considered time interval, treatment was extended to older patients and with a higher level of baseline disability. In fact, both the patients’ mean age and mean value of baseline mRS score showed a statistically significant increase over time, respectively from 66.3 to 75.51 years (*p = 0.006*) and from 0.22 to 1.22 (*p = 0.000*) (Table 1).

**Fig. 2:**
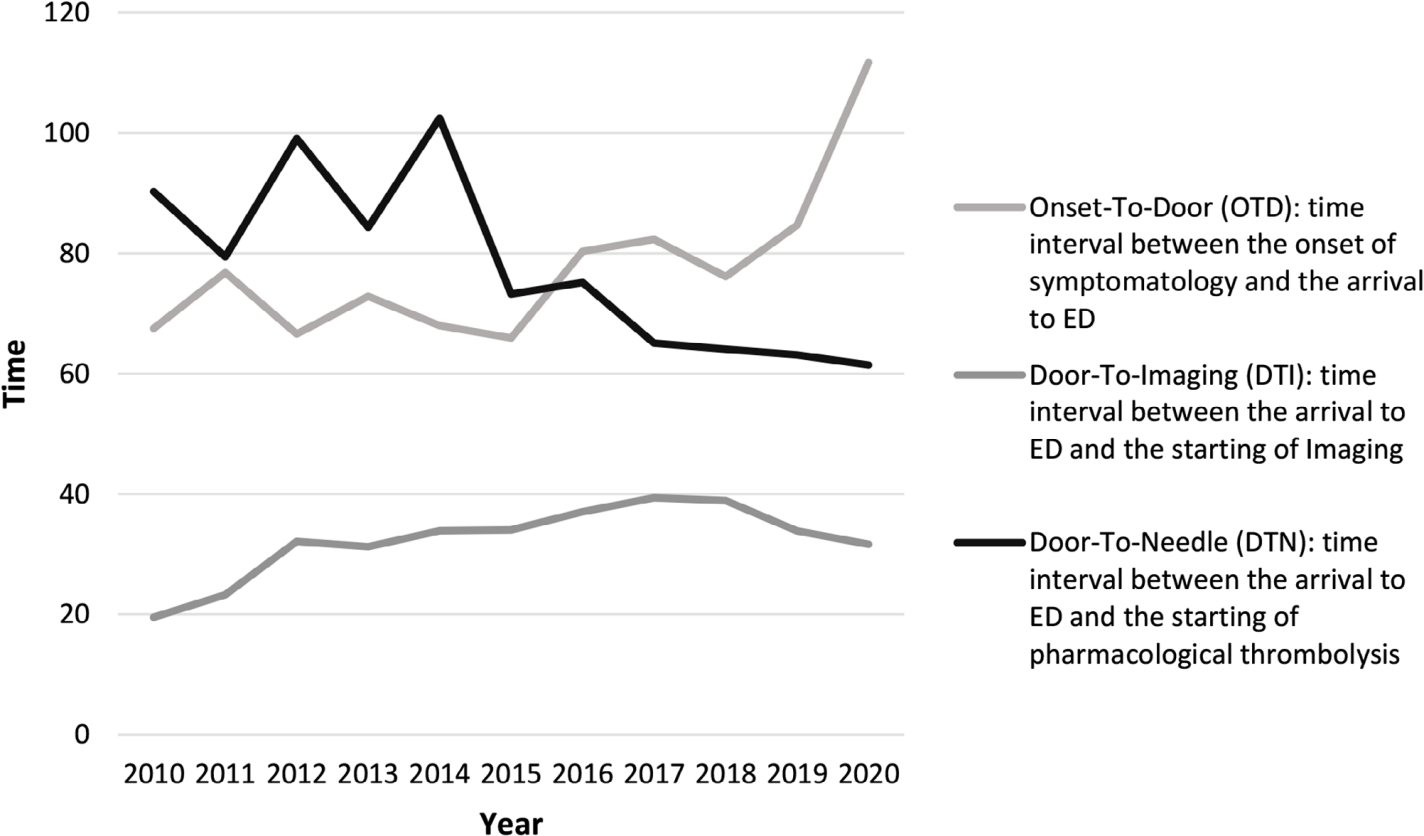
Onset-to-door (OTD), door-to-imaging (DTI), door-to-needle (DTN) annual trend

**Fig. 3:**
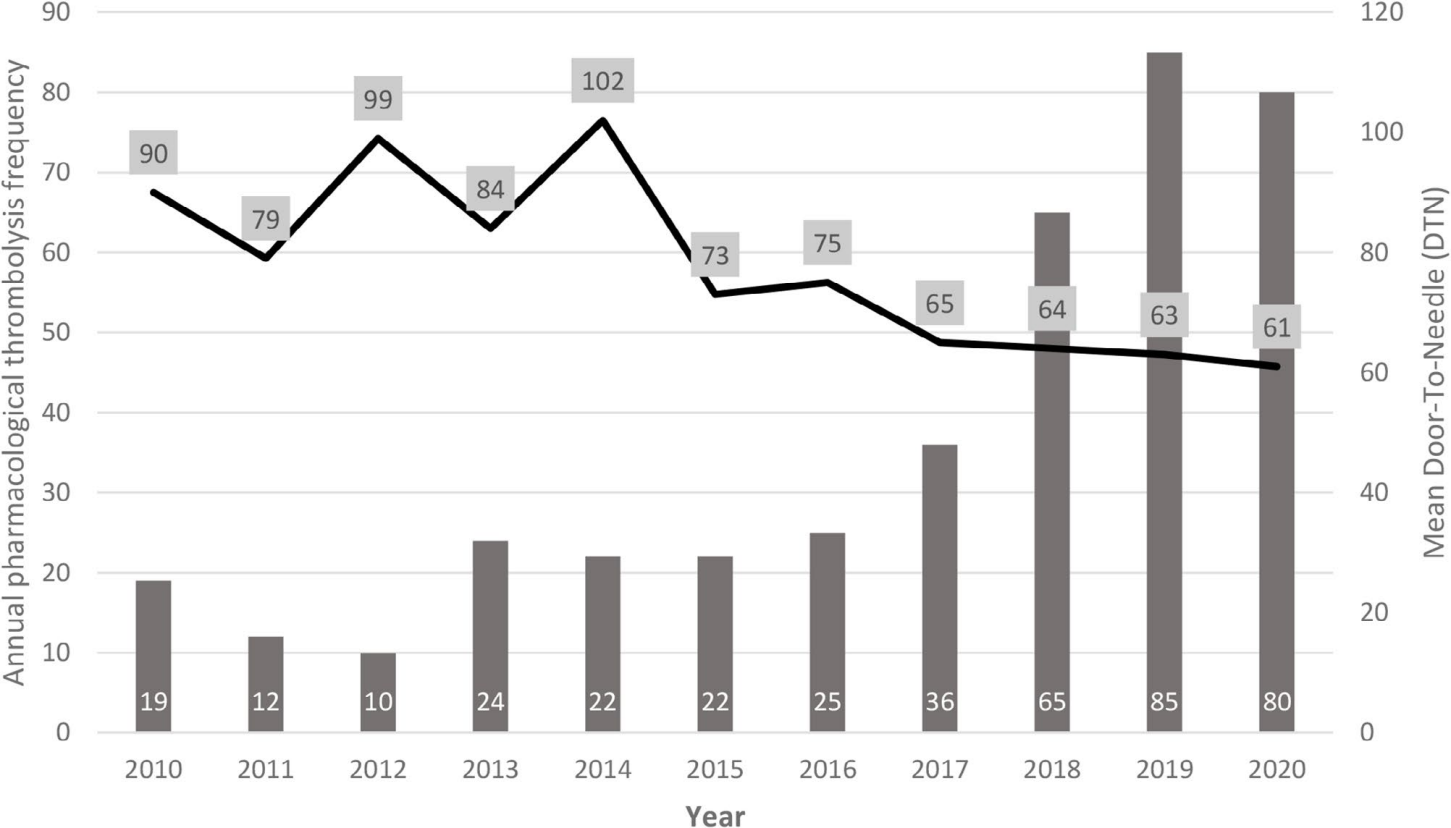
Annual trend of door-to-needle (DTN) in relation to the annual pharmacological thrombolysis volume

As for pre-hospital and hospital process indicators, OTD, and DTI time trends showed a substantial stability over time, while a statistically significant reduction over the years was found for DTN, going from 90 min to 61 min average value (*p = 0.000*) (Table 1; Fig. 2) and constantly decreasing from 2016 to 2020 versus the increasing annual thrombolysis volume (Fig. 3). The average reduction in DTN also led to an increase in the number of thrombolysis performed within the “golden hour”, according to the international target [16]. Indeed, although the target of 85% of patients treated within 60 min [17] was not reached, the improvements were constant, so that in 2019 more than 50% of patients were treated within 60 min (35% of them within 45 min) and in 2020 the treatments performed within 60 min amounted to 62% (27% of them within 45 min). The clinical outcome indicators 3 m-mRS and NHISS remained substantially unchanged over the decade covered by the study (Table 1).

The results of the comparison between the pre-pandemic (March-December 2019) and the pandemic (March-December 2020) periods are reported in Table 2. The number of patients remained almost unchanged (66 vs. 69), but with a significantly higher baseline disability (mRS = 1.18 vs. 0.72, *p = 0.048*). Moreover, in contrast to the trend in the considered decade, the OTD (pre-hospital pathway’s phase) increased from 88.13 to 118.48 min, although without a statistically significant difference (*p = 0.197*). On the contrary, both the other hospital process indicators DTI and DTN remained substantially unchanged, as well as the clinical outcome indicators 3 m-mRS, NHISS and 24 h-NHISS.

**Table 2:** Stroke pathway performance pre-post COVID-19 pandemic

		Pre-Covid (Mar-Dec 2019)	Covid (Mar-Dec 2020)	Total	*p*	
Number of patients		69	66	135		
%		51.1	48.9	100.0		
Age at stroke onset	Mean	75.75	75.00	75.39	*0.761*	
	Std. Deviation	14.498	14.061	14.239		
Onset-to-Door (OTD)	Mean	88.13	118.48	102.22	*0.197*	
	Std. Deviation	78.844	171.927	130.871		
Door-to-Imaging CT/MR (DTI)	Mean	32.93	32.66	32.81	*0.956*	
	Std. Deviation	20.605	31.773	26.012		
Door-to-Needle (DTN)	Mean	61.49	63.44	62.44	*0.673*	
	Std. Deviation	26.553	26.902	26.642		
Modified Ranking Scale (mRS) (Baseline)	Mean	0.72	1.18	0.95	***0.048***	
	Std. Deviation	1.162	1.499	1.351		
NIHSS (Baseline)	Mean	10.32	10.84	10.52	*0.725*	
	Std. Deviation	7.329	7.693	7.442		
NIHSS (24 h)	Mean	5.24	5.57	5.33	*0.852*	
	Std. Deviation	6.715	7.208	6.809		
Modified Ranking Scale after 3 months (mRS 3 m)	Mean	2.76	2.44	2.62	*0.520*	
	Std. Deviation	2.257	2.576	2.395	*0.761*	

## Discussion

The study shows a considerable improvement of the assessed Stroke Pathway over the years both in terms of efficiency and effectiveness. As for activity volumes and patients’ characteristics, the number of stroke cases treated with pharmacological thrombolysis increased over the years, as well as their mean age and baseline disability level. This can be traced back to the change over time, according to guidelines [1, 8], of the inclusion criteria, in particular to the extension of the time span for pharmacological thrombolysis from 3 to 4.5 h after the symptoms onset.

In addition, the incremental number of thrombolysis also led to the improvement of the Pathway’s performances, in particular those related to the hospital stroke management phase. The process analysis, indeed, highlighted a significant reduction of the DTN value, i.e. the time interval between the arrival to ED and the starting of pharmacological thrombolysis, parallel to the increased annual number of cases treated. This data reveals the learning curve of the system, which, in a virtuous circle, improves its parameters with higher treatment volumes. To this regard, the relationship between case volume and performance is widely reported in the scientific literature, for several clinical areas [18–25] including stroke treatment [26, 27]. In spite of the DTN reduction, the DTI value showed an increase over time, albeit without statistical significance. It could be attributable to contextual variables (e.g. logistical/organizational factors, patient flows) worthy of future investigations aimed at analyzing and understanding the reasons underlying the pathway’s performance, which the present study aimed to assess. Anyway, the DTI increase observed over time did not negatively affect neither DTN, despite being its sub-stage, nor clinical outcome.

Moreover, the lower restriction in patient selection related to the compliance of the Stroke Pathway with guidelines [1, 8] did not worsen the long-term prognosis. In fact, despite an increasing mean value of baseline disability, measured by the mRS score, over the years, the degree of disability 3 months after the ischemic event onset, as expressed by the 3 m-mRS, remained stable. It was probably due to a balancing effect linked to improvements in the acute management, in particular DTN reduction, and therefore in the effectiveness of treatment. The relationship between the reduction in time metrics and a higher chance of getting a good neurological recovery at 3 months is also reported by scientific literature [28].

Unlike DTN, the time interval between the onset of symptomatology and the arrival to ED (OTD) did not change significantly over the years. The OTD stability can be traced back to a level of patients/caregivers’ education still not enough to timely recognize stroke signs and symptoms resulting in a delayed request for emergency healthcare. Some Authors recently underlined the need for enhancing patient awareness on timely emergence medical services usage at stroke onset by strengthening publicity and educational activities [29, 30].

It can also be said that, despite the onset of the COVID-19 pandemic and its negative impact on Italian healthcare system [12], the Stroke Pathway has proven effective both in terms of hospital processes and clinical outcomes for patients undergoing pharmacological thrombolysis. In fact, the comparison of the pre-pandemic (March-December 2019) and the pandemic periods (March-December 2020) showed a number of positive elements. Concerning hospital processes, both the DTI and DTN scores did not change significantly. As for patient outcomes, although a higher baseline disability level (mRS score), the long-term disability (3 m-mRS) score did not show a statistically significant change, except for a slight improvement. These results show the resilience, during the emergency phase, of the Stroke Pathway - especially of the hospital phase - and its positive impact on outcomes, given that the long-term disability level didn’t get worse. These data are in line with previous studies reporting the Stroke Pathway effectiveness and underlining its importance as a crucial clinical governance tool to improve patient clinical management [31–36]. On the other hand, the pre-post COVID-19 comparison showed an OTD increase, although not statistically significant. This data reveals the impact of the pandemic on the pre-hospital phase of the Stroke Pathway, unlike the hospital one. Indeed, it could be related to the hesitancy of people to go to ED or alert the emergency medical service [12] up to underestimate the stroke signs and symptoms, as also confirmed by the higher baseline disability level (mRS score).

This study has some weaknesses and strengths. Among weaknesses, there is the limited number of eligible patients, which made it more difficult to achieve statistical significance for all the variables considered. Among strengths, there is the use of data obtained from the SITS-MOST registry [11], which is a world-leading platform for stroke data, also used for extensive international studies for its completeness and systematicity. Moreover, there is an evident internal consistency of data over the 10 years range considered in terms of guidelines’ targets pursuit [1, 8, 16].

The results of the retrospective assessment of the Stroke Pathway performances highlighted its positive impact both on hospital processes, although the latter can be further improved, and patients’ outcomes, even during the COVID-19 pandemic. Moreover, the analysis showed the need to identify and implement improvement actions for the pre-hospital phase.

What the main study implications? As far as clinical management is concerned, the first objective is to improve the hospital stroke management phase, through a change of the Stroke Pathway with the thrombolysis starting in the diagnostic imaging department in order to further reduce the DTN score and achieve the international guidelines standards [8]. The second objective is to promote health communication actions aimed at the general population, from specialists up to patients/caregivers, through information/education campaigns easily accessible to the public. The last should provide simple essential information to recognize stroke signs and symptoms in order to reduce the time between the onset of symptoms and the arrival to ED. In this regard, in 2018 the Emilia-Romagna Region, launched, through the Regional Health Website, the “I see, I recognize, I call” initiative, an awareness campaign for the early and timely recognition of stroke symptoms. The dissemination of information reaches users through an illustrative video available on the website and the distribution of leaflets/brochures to health providers and patients/citizens [37]. Other actions should be promoted and implemented to strengthen communication and make it more effective, such as the engagement of general practitioners and institutional health education initiatives/events involving all the stakeholders also through the use of social media. As for research, this study paves the way for a multicentric study, on a larger sample, to confirm the results of this retrospective assessment and better investigate the analyzed variables. Moreover, future investigation will be useful to analyze the context variables, internal and external to the Hospital, which allowed the standards to be achieved and maintained in such a challenging contingency as the COVID-19 pandemic.

## Conclusion

Performance assessment is a key element in the process of continuous healthcare quality improvement. Data collection and usability is an essential pre-requisite for the systematic monitoring activity and the identification of improvement actions. Clinical registries serve as a useful tool to this aim, allowing for structured data entry and benchmarking.

Our study, based on the SIST-MOST registry data, showed a gradual improvement, over 10 years of observation, of the Stroke Pathway, both in terms of healthcare processes and patients’ outcomes, and its resilience during the COVID-19 emergency. The analysis also highlighted the need of improvement actions for both hospital and pre-hospital phases to align with international goals. Further studies investigating the reasons for achieving and mantaining the Stroke Pathway performance levels may allow the identification of the specific changes still to be made in order to purse the continuous improvement of the Pathway efficiency and effectiveness.

## Data Availability

All data analyzed in this study are recorded in the SITS-MOST registry, a world-leading platform of all patients undergoing pharmacological thrombolysis.

## Declarations

Since centres connected to SITS-MOST registry use thrombectomy in clinical practice, consent for the treatment is granted by clinical routine procedures. Patients receive written information about the registry and its purpose and they may decline to be included in the registry. Taking data from that anonymous register, for the present study informed consent was waived by the same ethics committee that approved the study (Ethical Committee of the Romagna Scientific Institute).

## References

[1] Italian Stroke Association-Associazione Italiana Ictus. Linee Guida SPREAD Stroke Prevention And Educational Awareness Diffusion VIII Edizione. Ictus Cerebrale: linee guida italiane di prevenzione e trattamento. Available online at: https://isa-aii.com/linee-guida-spread-viii-edizione/ (last access: 27.01.2023)].

[2] Stornello M, Sanzaro E. Epidemiologia e classificazione. Quaderni dell’Italian Journal of Medicine. Aggiornamenti in tema di malattia cerebrovascolare: prevenzione, terapia e riabilitazione 2020; 8(2):4–6. Available online at: https://www.italjmed.org/index.php/ijm/article/view/itjm.q.2020.2/1273 (last access: 25/01/2023).

[3] GBD 2016 Stroke Collaborators (2019). Global, regional, and national burden of Stroke, 1990–2016: a systematic analysis for the global burden of Disease Study 2016. Lancet Neurol.

[4] GBD 2019 Stroke Collaborators (2021). Global, regional, and national burden of Stroke and its risk factors, 1990–2019: a systematic analysis for the global burden of Disease Study 2019. Lancet Neurol.

[5] Sultan S, Elkind MS (2013). The growing problem of Stroke among young adults. Curr Cardiol Rep.

[6] Smajlović D (2015). Strokes in young adults: epidemiology and prevention. Vasc Health Risk Manag.

[7] O’Donnell MJ, Xavier D, Liu L, Zhang H, Chin SL, Rao-Melacini P, Rangarajan S, Islam S, Pais P, McQueen MJ, Mondo C, Damasceno A, Lopez-Jaramillo P, Hankey GJ, Dans AL, Yusoff K, Truelsen T, Diener HC, Sacco RL, Ryglewicz D, Czlonkowska A, Weimar C, Wang X, Yusuf S (2010). INTERSTROKE investigators. Risk factors for ischaemic and intracerebral haemorrhagic Stroke in 22 countries (the INTERSTROKE study): a case-control study. Lancet.

[8] Powers WJ, Rabinstein AA, Ackerson T, Adeoye OM, Bambakidis NC, Becker K, Biller J, Brown M, Demaerschalk BM, Hoh B, Jauch EC, Kidwell CS, Leslie-Mazwi TM, Ovbiagele B, Scott PA, Sheth KN, Southerland AM, Summers DV, Tirschwell DL (2019). Guidelines for the early management of patients with Acute ischemic Stroke: 2019 update to the 2018 guidelines for the early management of Acute ischemic Stroke: a Guideline for Healthcare professionals from the American Heart Association/American Stroke Association. Stroke.

[9] Ministero della Salute. Decreto Ministeriale 2 aprile 2015 n. 70. Regolamento recante definizione degli standard qualitativi, strutturali, tecnologici e quantitativi relativi all’assistenza ospedaliera. Gazzetta Ufficiale 4 giugno 2015, n. 127. Available online at: https://www.camera.it/temiap/2016/09/23/OCD177-2353.pdf (last access: 27.01.2023).

[10] Rotter T, Kinsman L, James E, Machotta A, Willis J, Snow P, Kugler J (2012). The effects of clinical pathways on professional practice, patient outcomes, length of stay, and hospital costs: Cochrane systematic review and meta-analysis. Eval Health Prof.

[11] Toni D, Lorenzano S, Puca E, Prencipe M (2006). The SITS-MOST registry. Neurol Sci.

[12] Frisullo G, Brunetti V, Di Iorio R, Broccolini A, Caliandro P, Monforte M, Morosetti R, Piano C, Pilato F, Calabresi P, Della Marca G, STROKE, TEAM Collaborators (2020). Effect of lockdown on the management of ischemic Stroke: an Italian experience from a COVID hospital. Neurol Sci.

[13] Evenson KR, Foraker RE, Morris DL, Rosamond WD (2009). A comprehensive review of prehospital and in-hospital delay times in acute Stroke care. Int J Stroke.

[14] Wilson JT, Hareendran A, Grant M, Baird T, Schulz UG, Muir KW, Bone I (2002). Improving the assessment of outcomes in Stroke: use of a structured interview to assign grades on the modified Rankin Scale. Stroke.

[15] National Institute of Neurological Disorders and Stroke. NIH Stroke Scale. Available online at: https://www.stroke.nih.gov/documents/NIH_Stroke_Scale_Booklet_508C.pdf (last access: 27.01.2023).

[16] Kim JT, Fonarow GC, Smith EE, Reeves MJ, Navalkele DD, Grotta JC, Grau-Sepulveda MV, Hernandez AF, Peterson ED, Schwamm LH, Saver JL (2017). Treatment with tissue plasminogen activator in the Golden Hour and the shape of the 4.5-Hour time-benefit curve in the National United States get with the guidelines-Stroke Population. Circulation.

[17] Rajan SS, Decker-Palmer M, Wise J, Dao T, Salem C, Savitz SI (2021). Beneficial effects of the 30-minute door-to-needle time standard for alteplase administration. Ann Clin Transl Neurol.

[18] Yao R, Yan M, Liang Q, Wang H, Liu Z, Li F, Zhang H, Li K, Sun F (2022). Clinical efficacy and learning curve of posterior percutaneous endoscopic cervical laminoforaminotomy for patients with cervical spondylotic radiculopathy. Med (Baltim).

[19] Mirza MZ, Olson SL, Panthofer AM, Matsumura JS, Williams SK (2022). Surgeon learning curve and clinical outcomes of minimally invasive anterior lumbar Interbody Fusion with posterior percutaneous instrumentation. J Am Acad Orthop Surg Glob Res Rev.

[20] Wassef AWA, Rodes-Cabau J, Liu Y, Webb JG, Barbanti M, Muñoz-García AJ, Tamburino C, Dager AE, Serra V, Amat-Santos IJ, Alonso Briales JH, San Roman A, Urena M, Himbert D, Nombela-Franco L, Abizaid A, de Brito FS, Ribeiro HB, Ruel M, Lima VC, Nietlispach F, Cheema AN (2018). The learning curve and Annual Procedure volume standards for Optimum outcomes of Transcatheter aortic valve replacement: findings from an International Registry. JACC Cardiovasc Interv.

[21] Russo MJ, McCabe JM, Thourani VH, Guerrero M, Genereux P, Nguyen T, Hong KN, Kodali S, Leon MB (2019). Case volume and outcomes after TAVR with balloon-expandable prostheses: insights from TVT Registry. J Am Coll Cardiol.

[22] Mihai R, Donatini G, Vidal O, Brunaud L (2019). Volume-outcome correlation in adrenal surgery-an ESES consensus statement. Langenbecks Arch Surg.

[23] Chua ME, Ming JM, Kim JK, Degheili J, Santos JD, Farhat WA (2019). Competence in and learning curve for Pediatric Renal Transplant using cumulative Sum analyses. J Urol.

[24] Lal BK, Mayorga-Carlin M, Kashyap V, Jordan W, Mukherjee D, Cambria R, Moore W, Neville RF, Eckstein HH, Sahoo S, Macdonald S, Sorkin JD (2022). Learning curve and proficiency metrics for transcarotid artery revascularization. J Vasc Surg.

[25] Barac YD, Loungani RS, Sabulsky R, Carr K, Zwischenberger B, Glower DD (2022). Sustained results of robotic mitral repair in a lower volume center with extensive minimally invasive mitral repair experience. J Robot Surg.

[26] de Castro-Afonso LH, Nakiri GS, Fornazari VR, Abud TG, Monsignore LM, Pazuello GB, Dias FA, Martins-Filho RKDV, Camilo MR, Aléssio-Alves FF, Fábio SC, Pazin-Filho A, Pontes-Neto OM, Abud DG (2021). Performance evolution over 645 acute Stroke thrombectomies in a public Brazilian healthcare institution. Int J Stroke.

[27] Cai Q, Zhu Y, Huang X, Xiao L, Gu M, Wang P, Zhang C, Chen J, Hu W, Wang G, Sun W (2021). Learning curve for endovascular treatment of anterior circulation large vessel occlusion at a single Center. Front Neurol.

[28] Nilanont Y, Chanyagorn P, Shukij K, Pengtong W, Kongmuangpuk M, Wongmayurachat K, Nittayaboon K, Wongsawat Y, Sirovetnukul R, Chakorn T, Riyapan S, Kaveeta C, Chotik-Anuchit S, Tongdee T, Thabmontian P, Saeheng P, Nopmaneejumruslers C, Vamvanij V (2022). Comparing performance measures and clinical outcomes between mobile Stroke units and usual care in underserved areas. Neurol Sci.

[29] Lu YX, Li SD, Shan GL, Peng B (2021). Association between intention to call EMS in Stroke patients and level of hospital classification and emergency medical service usage among Stroke patients in China. Neurol Neurochir Pol.

[30] Zhong X, Wang J, He L, Xu R (2020). Recognition of stroke-related knowledge among community residents and the improvement after intensive health education: a cross-sectional study. BMC Neurol.

[31] de Belvis AG, Lohmeyer FM, Barbara A, Giubbini G, Angioletti C, Frisullo G, Ricciardi W, Specchia ML (2019). Ischemic Stroke: clinical pathway impact. Int J Health Care Qual Assur.

[32] Threlkeld ZD, Kozak B, McCoy D, Cole S, Martin C, Singh V (2017). Collaborative interventions reduce time-to-thrombolysis for Acute ischemic Stroke in a Public Safety Net Hospital. J Stroke Cerebrovasc Dis.

[33] Schmidt A, Heroum C, Caumette D, Le Lay K, Bénard S (2015). Acute ischemic Stroke (AIS) patient management in French Stroke units and impact estimation of thrombolysis on care pathways and associated costs. Cerebrovasc Dis.

[34] Willeit J, Geley T, Schöch J, Rinner H, Tür A, Kreuzer H, Thiemann N, Knoflach M, Toell T, Pechlaner R, Willeit K, Klingler N, Praxmarer S, Baubin M, Beck G, Berek K, Dengg C, Engelhardt K, Erlacher T, Fluckinger T, Grander W, Grossmann J, Kathrein H, Kaiser N, Matosevic B, Matzak H, Mayr M, Perfler R, Poewe W, Rauter A, Schoenherr G, Schoenherr HR, Schinnerl A, Spiss H, Thurner T, Vergeiner G, Werner P, Wöll E, Willeit P, Kiechl S (2015). Thrombolysis and clinical outcome in patients with Stroke after implementation of the Tyrol Stroke pathway: a retrospective observational study. Lancet Neurol.

[35] Cloud G, Hoffman A, Rudd A (2013). Intercollegiate Stroke Working Party. National sentinel Stroke audit 1998–2011. Clin Med (Lond).

[36] Taylor WJ, Wong A, Siegert RJ, McNaughton HK (2006). Effectiveness of a clinical pathway for acute Stroke care in a district general hospital: an audit. BMC Health Serv Res.

[37] Emilia-Romagna, Region. I see, I recognize, I call. Available online at: https://salute.regione.emilia-romagna.it/campagne/ictus-vedo-riconosco-chiamo (last access: 14.02.2023).

